# *trans*-Bis(2,2′-di­pyridyl­amine-κ^2^ *N*,*N*′)­bis­(1,1,3,3-tetra­cyano-2-eth­oxy­propenido-κ*N*)copper(II)

**DOI:** 10.1107/S2414314622011804

**Published:** 2022-12-23

**Authors:** Yaakoub Saadallah, Zouaoui Setifi, David K. Geiger, Mohammed Hadi Al-Douh, Achouak Satour, Fatima Setifi

**Affiliations:** a Laboratoire de Chimie, Ingénierie Moléculaire et Nanostructures (LCIMN), Université Ferhat Abbas Sétif 1, Sétif 19000, Algeria; b Département de Technologie, Faculté de Technologie, Université 20 Août 1955-Skikda, BP 26, Route d’El-Hadaiek, Skikda 21000, Algeria; c Department of Chemistry, SUNY-College at Geneseo, Geneseo, NY 14454, USA; d Chemistry Department, Faculty of Science, Hadhramout University, Mukalla, Hadhramout, Yemen; Goethe-Universität Frankfurt, Germany

**Keywords:** crystal structure, copper(II), 2,2′-di­pyridyl­amine (dpa), 1,1,3,3-tetra­cyano-2-eth­oxy­propenide (tcnoet)

## Abstract

The title compound, [Cu(C_9_H_5_N_4_O)_2_(C_10_H_9_N_3_)_2_], was synthesized solvothermally. The complex exhibits a distorted octa­hedral coordination geometry. The Cu^II^ atom is located on an inversion centre. The distorted octahedral CuN_6_ coordination sphere is composed of bidentate 2,2′-dipyrid­ylamine in the equatorial sites while the axial sites are occupied by 1,1,3,3-tetra­cyano-2-eth­oxy­propenide ligands. In the crystal, N—H⋯N hydrogen bonding results in chains parallel to [010].

## Abstract 2

The title complex exhibits a distorted octa­hedral coordination geometry. The CuN_6_ coordination sphere is composed of bidentate 2,2′-dipyrid­yl)amine in the equatorial sites while the axial sites are occupied by 1,1,3,3-tetra­cyano-2-eth­oxy­propenide ligands. In the crystal, N—H⋯N hydrogen bonding results in chains parallel to [010].

## Graphical abstract

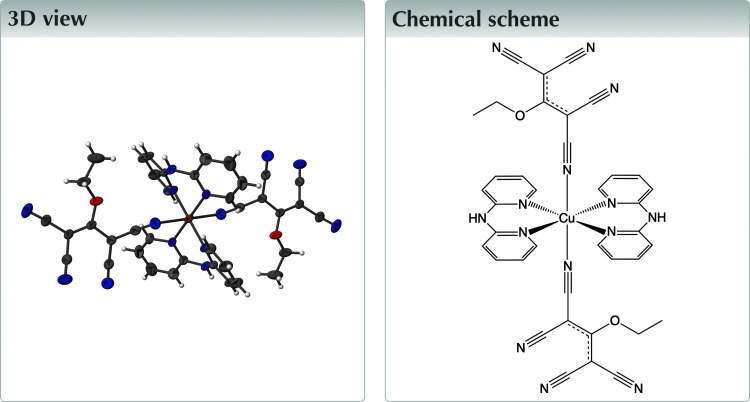

## Structure description

Anionic polynitrile ligands are of inter­est because of their ability to act as bridging ligands with different coordination modes to generate many different topologies by functioning alone or in combination with other neutral co-ligands (Miyazaki *et al.*, 2003 ▸; Benmansour *et al.*, 2008 ▸, 2010 ▸, 2012 ▸; Setifi *et al.*, 2013 ▸; Dmitrienko *et al.*, 2020 ▸). In view of this coordinating ability, these ligands have also been explored for their utility in developing materials capable of magnetic exchange coupling (Yuste *et al.*, 2009 ▸; Atmani *et al.*, 2008 ▸). As a part of our continuing studies of the structural and magnetic properties of Cu^II^ complexes containing both polynitrile and polypyridyl units (Setifi *et al.*, 2006 ▸, 2007 ▸, 2009 ▸, 2014 ▸; Addala *et al.*, 2015 ▸), we report here the synthesis and the crystal and mol­ecular structure of a new mononuclear compound based on 2,2′-di­pyridyl­amine (dpa) as co-ligand and 1,1,3,3-tetra­cyano-2-eth­oxy­propenide (tcnoet) as ligands.

**Figure 1:**
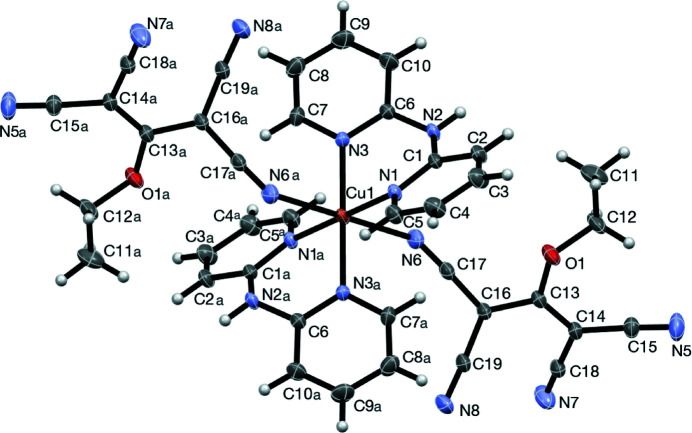
View of the title compound showing the atom-labelling scheme. Anisotropic displacement parameters of non-H atoms are drawn at the 30% probability level. [Symmetry code: (*a*) −*x* + 1, −*y* + 1, −*z* + 1.]

The title compound exhibits a distorted octa­hedral coordination environment, as expected for a six-coordinate, *d* ^9^ coordination complex due to the Jahn–Teller effect (see Table 1 ▸). The mol­ecular geometry and atom-labelling scheme are represented in Fig. 1 ▸. The Cu^II^ ion is located on an inversion centre. The bidentate dpa ligands occupy equatorial sites, with coordinating tcnoet ligands in the axial sites. The Cu—N6 bond length compares well with those reported for other Cu^II^ complexes with axially coordinated tcnoet ligands (Thetiot *et al.*, 2003 ▸; Addala *et al.*, 2015 ▸).

**Figure 2:**
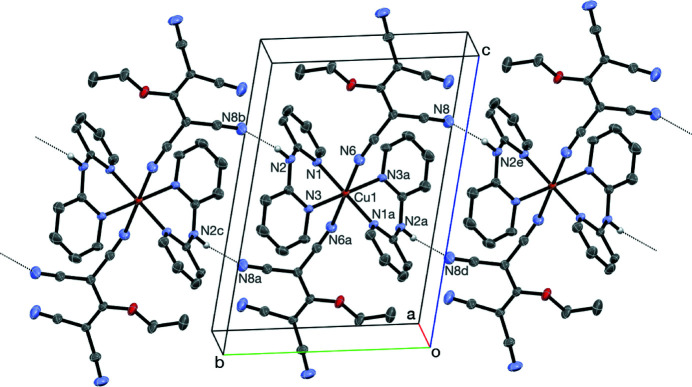
Partial packing diagram showing the N—H⋯N hydrogen-bonding inter­actions. Only H atoms involved in the inter­molecular inter­actions are shown. [Symmetry codes: (*a*) −*x* + 1, −*y* + 1, −*z* + 1; (*b*) *x*, *y* + 1, *z*; (*c*) −*x* + 1, −*y* + 2, −*z* + 1; (*d*) −*x* + 1,-*y*, −*z* + 1; (*e*) *x*, *y* − 1, *z*.]

The extended structure exhibits an N2—H2⋯N8 hydrogen-bonding network (Table 2 ▸), resulting in chains running parallel to [010], as seen in Fig. 2 ▸. Intra- and inter­molecular C—H⋯N hydrogen bonds are also observed (Table 2 ▸).

## Synthesis and crystallization

The title compound was synthesized solvothermally under autogenous pressure using a mixture of copper(II) sulfate penta­hydrate (25 mg, 0.1 mmol), 2,2′-di­pyridyl­amine (34 mg, 0.2 mmol) and potassium 1,1,3,3-tetra­cyano-2-eth­oxy­propenide (45 mg, 0.2 mmol) in water-methanol (3:1 *v*/*v*, 20 ml). The mixture was sealed in a Teflon-lined autoclave and held at 438 K for 2 d, and then cooled to ambient temperature at a rate of 10 K per hour (yield 42%). Green blocks of the title complex suitable for single-crystal X-ray diffraction were selected directly from the synthesized product.

## Refinement

Crystal data, data collection and structure refinement details are summarized in Table 3 ▸.

## Supplementary Material

Crystal structure: contains datablock(s) global, I. DOI: 10.1107/S2414314622011804/bt4129sup1.cif

Structure factors: contains datablock(s) I. DOI: 10.1107/S2414314622011804/bt4129Isup2.hkl

CCDC reference: 2225624

Additional supporting information:  crystallographic information; 3D view; checkCIF report

## Figures and Tables

**Table 1:** Selected geometric parameters (Å, °)

Cu1—N1	2.0196 (13)	Cu1—N6	2.4828 (16)
Cu1—N3	2.0196 (13)		
			
N1—Cu1—N3^i^	94.54 (5)	N3—Cu1—N6	92.15 (6)
N1—Cu1—N6	91.81 (6)		

Symmetry code: (i) 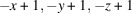 .

**Table 2:** Hydrogen-bond geometry (Å, °)

*D*—H⋯*A*	*D*—H	H⋯*A*	*D*⋯*A*	*D*—H⋯*A*
N2—H2⋯N8^ii^	0.85 (2)	2.18 (2)	3.020 (2)	171.2 (19)
C7—H7⋯N5^iii^	0.93	2.43	3.234 (2)	145
C10—H10⋯N8^ii^	0.93	2.62	3.385 (3)	140
C12—H12*A*⋯N5	0.97	2.64	3.404 (3)	136

Symmetry codes: (ii) 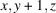 ; (iii) 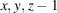 .

**Table 3:** Experimental details

Crystal data	
Chemical formula	[Cu(C_9_H_5_N_4_O)_2_(C_10_H_9_N_3_)_2_]
*M*_r_	776.28
Crystal system, space group	Triclinic, *P* 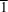
Temperature (K)	300
*a*, *b*, *c* (Å)	7.5101 (3), 9.2232 (4), 13.6405 (6)
α, β, γ (°)	99.068 (1), 98.864 (1), 93.139 (1)
*V* (Å^3^)	918.86 (7)
*Z*	1
Radiation type	Mo *K*α
μ (mm^−1^)	0.65
Crystal size (mm)	0.40 × 0.10 × 0.06

Data collection	
Diffractometer	Oxford Diffraction Xcalibur CCD
Absorption correction	Multi-scan (*CrysAlis RED*; Oxford Diffraction, 2009 ▸)
*T*_min_, *T*_max_	0.481, 1.000
No. of measured, independent and observed [*I* > 2σ(*I*)] reflections	51758, 5649, 4388
*R*_int_	0.062
(sin θ/λ)_max_ (Å^−1^)	0.716

Refinement	
*R*[*F* ^2^ > 2σ(*F* ^2^)], *wR*(*F* ^2^), *S*	0.039, 0.094, 1.05
No. of reflections	5649
No. of parameters	255
H-atom treatment	H atoms treated by a mixture of independent and constrained refinement
Δρ_max_, Δρ_min_ (e Å^−3^)	0.32, −0.44

Computer programs: *CrysAlis CCD* and *CrysAlis RED* (Oxford Diffraction, 2009 ▸), *SHELXS97* (Sheldrick, 2008 ▸), *SHELXL2014/7* (Sheldrick, 2015 ▸), *PLATON* (Spek, 2020 ▸), *Mercury* (Macrae *et al.*, 2020 ▸) and *publCIF* (Westrip, 2010 ▸).

## full crystallographic data

### Crystal data

**Table:**

[Cu(C_9_H_5_N_4_O)_2_(C_10_H_9_N_3_)_2_]	*Z* = 1
*M**_r_* = 776.28	*F*(000) = 399
Triclinic, *P*1	*D*_x_ = 1.403 Mg m^−^^3^
*a* = 7.5101 (3) Å	Mo *K*α radiation, λ = 0.71073 Å
*b* = 9.2232 (4) Å	Cell parameters from 937 reflections
*c* = 13.6405 (6) Å	θ = 2.6–28.1°
α = 99.068 (1)°	µ = 0.65 mm^−^^1^
β = 98.864 (1)°	*T* = 300 K
γ = 93.139 (1)°	Block, blue
*V* = 918.86 (7) Å^3^	0.40 × 0.10 × 0.06 mm

### Data collection

**Table:**

Oxford Diffraction X calibur CCD diffractometer	4388 reflections with *I* > 2σ(*I*)
Radiation source: Enhance (Mo) X-ray source	*R*_int_ = 0.062
ω scans	θ_max_ = 30.6°, θ_min_ = 2.5°
Absorption correction: multi-scan (CrysalisRed; Oxford Diffraction, 2009)	*h* = −10→10
*T*_min_ = 0.481, *T*_max_ = 1.000	*k* = −13→13
51758 measured reflections	*l* = −19→19
5649 independent reflections	

### Refinement

**Table:**

Refinement on *F*^2^	Primary atom site location: structure-invariant direct methods
Least-squares matrix: full	Hydrogen site location: mixed
*R*[*F*^2^ > 2σ(*F*^2^)] = 0.039	H atoms treated by a mixture of independent and constrained refinement
*wR*(*F*^2^) = 0.094	*w* = 1/[σ^2^(*F*_o_^2^) + (0.0294*P*)^2^ + 0.4397*P*] where *P* = (*F*_o_^2^ + 2*F*_c_^2^)/3
*S* = 1.05	(Δ/σ)_max_ < 0.001
5649 reflections	Δρ_max_ = 0.32 e Å^−^^3^
255 parameters	Δρ_min_ = −0.44 e Å^−^^3^
0 restraints	

### Special details

**Table:**

Geometry. All esds (except the esd in the dihedral angle between two l.s. planes) are estimated using the full covariance matrix. The cell esds are taken into account individually in the estimation of esds in distances, angles and torsion angles; correlations between esds in cell parameters are only used when they are defined by crystal symmetry. An approximate (isotropic) treatment of cell esds is used for estimating esds involving l.s. planes.
Refinement. All hydrogen atoms bonded to C atoms were positioned geometrically and treated as riding atoms, using C—H = 0.93 Å (aromatic), 0.96 Å (CH_3_) or 0.97 Å (CH_2_), and with *U*_iso_(H) = k*U*_eq_(C), where k = 1.5 for the methyl groups and 1.2 for all other H atoms bonded to C atoms. The H atom bonded to the amine N atom was refined freely, including its isotropic displacement parameter.

### Fractional atomic coordinates and isotropic or equivalent isotropic displacement parameters (Å^2^)

**Table:**

	*x*	*y*	*z*	*U*_iso_*/*U*_eq_	
Cu1	0.5	0.5	0.5	0.03141 (9)	
N1	0.67538 (19)	0.64977 (14)	0.59690 (10)	0.0313 (3)	
N2	0.4406 (2)	0.79956 (16)	0.62884 (10)	0.0345 (3)	
H2	0.413 (3)	0.872 (2)	0.6688 (16)	0.042 (5)*	
N3	0.37389 (19)	0.67548 (15)	0.46127 (10)	0.0313 (3)	
O1	0.33800 (19)	0.57177 (13)	0.88700 (10)	0.0459 (3)	
N5	0.2377 (3)	0.4862 (2)	1.13970 (13)	0.0691 (6)	
N6	0.3087 (3)	0.47360 (18)	0.63022 (12)	0.0506 (4)	
N7	0.0144 (3)	0.1256 (2)	0.91228 (16)	0.0691 (6)	
N8	0.3262 (3)	0.07139 (19)	0.75000 (14)	0.0630 (5)	
C1	0.6156 (2)	0.76147 (16)	0.65471 (11)	0.0301 (3)	
C2	0.7266 (3)	0.84429 (19)	0.73900 (13)	0.0416 (4)	
H2A	0.68	0.9168	0.781	0.05*	
C3	0.9045 (3)	0.8172 (2)	0.75871 (16)	0.0511 (5)	
H3	0.98	0.8703	0.8148	0.061*	
C4	0.9713 (3)	0.7099 (2)	0.69434 (16)	0.0487 (5)	
H4	1.0934	0.6934	0.7044	0.058*	
C5	0.8543 (2)	0.6287 (2)	0.61567 (14)	0.0394 (4)	
H5	0.8991	0.5557	0.5731	0.047*	
C6	0.3509 (2)	0.78931 (17)	0.53097 (12)	0.0318 (3)	
C7	0.2986 (3)	0.6766 (2)	0.36427 (13)	0.0403 (4)	
H7	0.3195	0.6006	0.315	0.048*	
C8	0.1940 (3)	0.7837 (2)	0.33556 (15)	0.0506 (5)	
H8	0.1454	0.7811	0.2683	0.061*	
C9	0.1619 (3)	0.8964 (3)	0.40891 (17)	0.0568 (6)	
H9	0.0872	0.9688	0.3918	0.068*	
C10	0.2409 (3)	0.9008 (2)	0.50707 (15)	0.0482 (5)	
H10	0.2217	0.9766	0.557	0.058*	
C11	0.3586 (4)	0.8295 (2)	0.9359 (2)	0.0680 (7)	
H11A	0.4881	0.8317	0.9499	0.102*	
H11B	0.3188	0.9096	0.979	0.102*	
H11C	0.321	0.839	0.8669	0.102*	
C12	0.2784 (4)	0.6878 (2)	0.95455 (17)	0.0560 (6)	
H12A	0.3177	0.676	1.0238	0.067*	
H12B	0.1475	0.6855	0.9424	0.067*	
C13	0.2780 (2)	0.43072 (17)	0.87862 (12)	0.0315 (3)	
C14	0.2105 (2)	0.37033 (18)	0.95387 (12)	0.0335 (3)	
C15	0.2291 (3)	0.4382 (2)	1.05654 (13)	0.0417 (4)	
C16	0.2916 (2)	0.34824 (18)	0.78366 (12)	0.0344 (4)	
C17	0.3030 (3)	0.42011 (19)	0.70040 (13)	0.0377 (4)	
C18	0.1052 (3)	0.2327 (2)	0.92985 (13)	0.0415 (4)	
C19	0.3079 (3)	0.1948 (2)	0.76699 (13)	0.0407 (4)	

### Atomic displacement parameters (Å^2^)

**Table:**

	*U*^11^	*U*^22^	*U*^33^	*U*^12^	*U*^13^	*U*^23^
Cu1	0.04112 (17)	0.02558 (14)	0.02394 (14)	0.00860 (11)	0.00108 (11)	−0.00461 (10)
N1	0.0365 (7)	0.0290 (6)	0.0257 (6)	0.0051 (5)	0.0044 (5)	−0.0037 (5)
N2	0.0445 (8)	0.0299 (7)	0.0270 (7)	0.0121 (6)	0.0069 (6)	−0.0056 (5)
N3	0.0402 (7)	0.0299 (6)	0.0231 (6)	0.0083 (6)	0.0039 (5)	0.0018 (5)
O1	0.0580 (8)	0.0292 (6)	0.0501 (8)	−0.0050 (6)	0.0255 (6)	−0.0082 (5)
N5	0.0958 (16)	0.0746 (14)	0.0308 (9)	−0.0078 (12)	0.0136 (9)	−0.0077 (9)
N6	0.0751 (12)	0.0427 (9)	0.0402 (9)	0.0118 (8)	0.0270 (8)	0.0065 (7)
N7	0.1046 (17)	0.0401 (10)	0.0621 (12)	−0.0155 (10)	0.0345 (12)	−0.0062 (9)
N8	0.1070 (16)	0.0381 (9)	0.0497 (10)	0.0243 (10)	0.0317 (11)	0.0004 (8)
C1	0.0411 (9)	0.0237 (7)	0.0246 (7)	0.0038 (6)	0.0062 (6)	0.0004 (6)
C2	0.0569 (11)	0.0287 (8)	0.0331 (9)	0.0042 (8)	0.0005 (8)	−0.0069 (7)
C3	0.0535 (12)	0.0390 (10)	0.0490 (11)	−0.0008 (9)	−0.0125 (9)	−0.0061 (8)
C4	0.0383 (10)	0.0434 (10)	0.0579 (12)	0.0025 (8)	−0.0034 (9)	0.0004 (9)
C5	0.0378 (9)	0.0376 (9)	0.0411 (9)	0.0069 (7)	0.0072 (7)	−0.0004 (7)
C6	0.0370 (8)	0.0295 (8)	0.0297 (8)	0.0084 (6)	0.0080 (6)	0.0029 (6)
C7	0.0500 (10)	0.0451 (10)	0.0246 (8)	0.0065 (8)	0.0035 (7)	0.0037 (7)
C8	0.0548 (12)	0.0627 (13)	0.0359 (10)	0.0155 (10)	0.0006 (9)	0.0162 (9)
C9	0.0601 (13)	0.0600 (13)	0.0559 (13)	0.0299 (11)	0.0059 (10)	0.0221 (10)
C10	0.0575 (12)	0.0427 (10)	0.0474 (11)	0.0248 (9)	0.0119 (9)	0.0064 (8)
C11	0.0748 (16)	0.0323 (10)	0.0917 (19)	−0.0020 (10)	0.0112 (14)	−0.0005 (11)
C12	0.0835 (16)	0.0299 (9)	0.0549 (12)	0.0032 (9)	0.0289 (11)	−0.0086 (8)
C13	0.0350 (8)	0.0281 (7)	0.0295 (8)	0.0034 (6)	0.0076 (6)	−0.0030 (6)
C14	0.0420 (9)	0.0317 (8)	0.0255 (7)	0.0056 (7)	0.0072 (7)	−0.0017 (6)
C15	0.0478 (10)	0.0447 (10)	0.0307 (9)	0.0008 (8)	0.0082 (8)	0.0005 (7)
C16	0.0458 (10)	0.0290 (8)	0.0298 (8)	0.0060 (7)	0.0140 (7)	0.0003 (6)
C17	0.0495 (10)	0.0309 (8)	0.0349 (9)	0.0077 (7)	0.0183 (8)	−0.0010 (7)
C18	0.0612 (12)	0.0335 (9)	0.0317 (9)	0.0058 (8)	0.0172 (8)	0.0009 (7)
C19	0.0586 (11)	0.0349 (9)	0.0308 (8)	0.0107 (8)	0.0166 (8)	0.0004 (7)

### Geometric parameters (Å, º)

**Table:**

Cu1—N1	2.0196 (13)	C4—C5	1.367 (3)
Cu1—N1^i^	2.0196 (13)	C4—H4	0.93
Cu1—N3^i^	2.0196 (13)	C5—H5	0.93
Cu1—N3	2.0196 (13)	C6—C10	1.400 (2)
Cu1—N6^i^	2.4828 (16)	C7—C8	1.363 (3)
Cu1—N6	2.4828 (16)	C7—H7	0.93
N1—C1	1.3374 (19)	C8—C9	1.383 (3)
N1—C5	1.359 (2)	C8—H8	0.93
N2—C6	1.386 (2)	C9—C10	1.372 (3)
N2—C1	1.386 (2)	C9—H9	0.93
N2—H2	0.85 (2)	C10—H10	0.93
N3—C6	1.339 (2)	C11—C12	1.484 (3)
N3—C7	1.358 (2)	C11—H11A	0.96
O1—C13	1.335 (2)	C11—H11B	0.96
O1—C12	1.436 (2)	C11—H11C	0.96
N5—C15	1.142 (2)	C12—H12A	0.97
N6—C17	1.149 (2)	C12—H12B	0.97
N7—C18	1.140 (3)	C13—C14	1.389 (2)
N8—C19	1.145 (2)	C13—C16	1.416 (2)
C1—C2	1.400 (2)	C14—C18	1.422 (2)
C2—C3	1.366 (3)	C14—C15	1.423 (2)
C2—H2A	0.93	C16—C19	1.413 (2)
C3—C4	1.385 (3)	C16—C17	1.413 (2)
C3—H3	0.93		
			
N1—Cu1—N1^i^	180.0	N3—C6—N2	119.59 (14)
N1—Cu1—N3^i^	94.54 (5)	N3—C6—C10	121.57 (16)
N1^i^—Cu1—N3^i^	85.46 (5)	N2—C6—C10	118.82 (15)
N1—Cu1—N3	85.46 (5)	N3—C7—C8	123.29 (17)
N1^i^—Cu1—N3	94.54 (5)	N3—C7—H7	118.4
N3^i^—Cu1—N3	180.00 (7)	C8—C7—H7	118.4
N1—Cu1—N6^i^	88.19 (6)	C7—C8—C9	118.40 (18)
N1^i^—Cu1—N6^i^	91.82 (6)	C7—C8—H8	120.8
N3^i^—Cu1—N6^i^	92.15 (6)	C9—C8—H8	120.8
N3—Cu1—N6^i^	87.85 (6)	C10—C9—C8	119.61 (18)
N1—Cu1—N6	91.81 (6)	C10—C9—H9	120.2
N1^i^—Cu1—N6	88.18 (6)	C8—C9—H9	120.2
N3^i^—Cu1—N6	87.85 (6)	C9—C10—C6	119.00 (18)
N3—Cu1—N6	92.15 (6)	C9—C10—H10	120.5
N6^i^—Cu1—N6	180.0	C6—C10—H10	120.5
C1—N1—C5	117.73 (14)	C12—C11—H11A	109.5
C1—N1—Cu1	120.69 (11)	C12—C11—H11B	109.5
C5—N1—Cu1	120.93 (11)	H11A—C11—H11B	109.5
C6—N2—C1	124.60 (14)	C12—C11—H11C	109.5
C6—N2—H2	113.2 (14)	H11A—C11—H11C	109.5
C1—N2—H2	113.6 (14)	H11B—C11—H11C	109.5
C6—N3—C7	117.92 (14)	O1—C12—C11	107.53 (18)
C6—N3—Cu1	121.18 (11)	O1—C12—H12A	110.2
C7—N3—Cu1	120.73 (11)	C11—C12—H12A	110.2
C13—O1—C12	122.75 (14)	O1—C12—H12B	110.2
C17—N6—Cu1	141.76 (15)	C11—C12—H12B	110.2
N1—C1—N2	119.24 (14)	H12A—C12—H12B	108.5
N1—C1—C2	121.84 (16)	O1—C13—C14	124.44 (14)
N2—C1—C2	118.89 (14)	O1—C13—C16	112.14 (15)
C3—C2—C1	119.08 (17)	C14—C13—C16	123.42 (15)
C3—C2—H2A	120.5	C13—C14—C18	120.00 (15)
C1—C2—H2A	120.5	C13—C14—C15	125.50 (16)
C2—C3—C4	119.39 (17)	C18—C14—C15	114.42 (16)
C2—C3—H3	120.3	N5—C15—C14	176.1 (2)
C4—C3—H3	120.3	C19—C16—C17	115.91 (14)
C5—C4—C3	118.66 (18)	C19—C16—C13	123.65 (16)
C5—C4—H4	120.7	C17—C16—C13	120.29 (15)
C3—C4—H4	120.7	N6—C17—C16	177.3 (2)
N1—C5—C4	122.93 (17)	N7—C18—C14	176.8 (2)
N1—C5—H5	118.5	N8—C19—C16	176.7 (2)
C4—C5—H5	118.5		
			
C5—N1—C1—N2	170.75 (15)	C6—N3—C7—C8	−3.4 (3)
Cu1—N1—C1—N2	−18.4 (2)	Cu1—N3—C7—C8	172.08 (16)
C5—N1—C1—C2	−7.2 (2)	N3—C7—C8—C9	−0.4 (3)
Cu1—N1—C1—C2	163.65 (13)	C7—C8—C9—C10	2.5 (4)
C6—N2—C1—N1	−33.7 (2)	C8—C9—C10—C6	−0.8 (3)
C6—N2—C1—C2	144.31 (17)	N3—C6—C10—C9	−3.1 (3)
N1—C1—C2—C3	4.7 (3)	N2—C6—C10—C9	175.5 (2)
N2—C1—C2—C3	−173.20 (18)	C13—O1—C12—C11	−174.33 (19)
C1—C2—C3—C4	0.8 (3)	C12—O1—C13—C14	−25.1 (3)
C2—C3—C4—C5	−3.5 (3)	C12—O1—C13—C16	155.17 (18)
C1—N1—C5—C4	4.4 (3)	O1—C13—C14—C18	162.61 (17)
Cu1—N1—C5—C4	−166.43 (16)	C16—C13—C14—C18	−17.7 (3)
C3—C4—C5—N1	0.9 (3)	O1—C13—C14—C15	−14.0 (3)
C7—N3—C6—N2	−173.51 (16)	C16—C13—C14—C15	165.63 (18)
Cu1—N3—C6—N2	11.1 (2)	O1—C13—C16—C19	153.95 (18)
C7—N3—C6—C10	5.1 (3)	C14—C13—C16—C19	−25.7 (3)
Cu1—N3—C6—C10	−170.32 (15)	O1—C13—C16—C17	−21.4 (2)
C1—N2—C6—N3	37.9 (2)	C14—C13—C16—C17	158.95 (18)
C1—N2—C6—C10	−140.78 (18)		

Symmetry code: (i) −*x*+1, −*y*+1, −*z*+1.

### Hydrogen-bond geometry (Å, º)

**Table:**

*D*—H···*A*	*D*—H	H···*A*	*D*···*A*	*D*—H···*A*
N2—H2···N8^ii^	0.85 (2)	2.18 (2)	3.020 (2)	171.2 (19)
C7—H7···N5^iii^	0.93	2.43	3.234 (2)	145
C10—H10···N8^ii^	0.93	2.62	3.385 (3)	140
C12—H12*A*···N5	0.97	2.64	3.404 (3)	136

Symmetry codes: (ii) *x*, *y*+1, *z*; (iii) *x*, *y*, *z*−1.
